# Association of long-term triglyceride-glucose index level and change with the risk of cardiometabolic diseases

**DOI:** 10.3389/fendo.2023.1148203

**Published:** 2023-03-30

**Authors:** Wenqi Xu, Haiyan Zhao, Lishu Gao, Lu Guo, Jianrong Liu, Haixia Li, Junyan Sun, Aijun Xing, Shuohua Chen, Shouling Wu, Yuntao Wu

**Affiliations:** ^1^ Department of Cardiology, Kailuan General Hospital, Tangshan, Hebei, China; ^2^ Graduate School, North China University of Science and Technology, Tangshan, Hebei, China; ^3^ Department of Endocrinology, Tangshan People's Hospital, Tangshan, Hebei, China

**Keywords:** triglyceride-glucose index, triglyceride-glucose index change, cardiometabolic diseases, cumulative effect, cohort study

## Abstract

**Objective:**

The triglyceride-glucose (TyG) index is considered as a pivotal factor for various metabolic, cardiovascular, and cerebrovascular diseases. However, there is currently a paucity of relevant studies on the association between long-term level and change of TyG-index and cardiometabolic diseases (CMDs) risk. We aimed to explore the risk of CMDs in relation to the long-term level and change of TyG-index.

**Methods:**

Based on the prospective cohort study, a total of 36359 subjects who were free of CMDs, had complete data of triglyceride (TG) and fasting blood glucose (FBG) and underwent four health check-ups from 2006 to 2012 consecutively were followed up for CMDs until 2021. The associations between long-term level and change of TyG-index and CMDs risk were assessed by Cox proportional hazards regression models to compute hazard ratios (HRs) and 95% confidence intervals (CIs). The TyG-index was calculated as ln [TG, mg/dL) × FBG, mg/dL)/2].

**Results:**

During the median observation period of 8 years, 4685 subjects were newly diagnosed with CMDs. In multivariable-adjusted models, a graded positive association was observed between CMDs and long-term TyG-index. Compared with the Q1 group, subjects with the Q2-Q4 group had increased progressively risk of CMDs, with corresponding HRs of 1.64(1.47-1.83), 2.36(2.13-2.62), 3.15(2.84-3.49), respectively. The association was marginally attenuated, after further adjustment for the baseline TyG level. In addition, compared with stable TyG level, both loss and gain in TyG level were associated with increased CMDs risk.

**Conclusions:**

Long-term elevated level and change of TyG-index are risk factors for the incident CMDs. Elevated TyG-index in the early stage remains to exert cumulative effects on the occurrence of CMDs even after accounting for the baseline TyG-index.

## Introduction

Cardiometabolic diseases (CMDs), encompassing type 2 diabetes mellitus (T2DM) and cardiovascular diseases (CVDs), remain the most predominant cause of permanent disability and mortality as a cluster of metabolic disorders on a global scale (1–3).

CMDs still impose a substantial burden on the medical and health industry, accounting for approximately 31% of total mortality worldwide, as identified by the latest World Health Organization (WHO) report (1). Early detection of groups at high risk and the recognition and control of risk factors, in particular modifiable factors, are therefore pivotal to preventing CMDs. The traditional risk factors, such as unhealthy living practices characterized by a lack of physical activity and poor diet (including smoking, alcohol consumption, and obesity), contribute to but cannot fully explain the increased risk of CMDs (4–6).

The existing robust epidemiological evidence implies that insulin resistance (IR), which has been recognized as an independent risk factor, contributes to the initiation and perpetuation of CMDs (7, 8). Nevertheless, the TyG-index, known as an alternative method for measuring IR (9–11), could be applied for clinical purposes and has shown a significant relation with IR (12, 13). Previous studies have demonstrated a positive correlation between the risk of developing CVD and the TyG-index (14). Moreover, a Korean cohort study with a 12-year follow-up reported that the TyG-index is regarded as a strong predictor for the development of T2DM among middle-aged and aged populations (15). Notably, however, measurements of the TyG-index in these related studies at a single time point might not be sufficient to indicate the long-term longitudinal effect of the TyG level on CMDs accurately. Moreover, both at home and abroad, the association between CMDs and the long-term dynamic changes in the TyG-index are scarce. Only one study, Wang et al. (16), has explored the association between CVD and change in the TyG-index thus far. However, the abovementioned research is inherently limited to a single event in CMDs and does not take into account the direction of change in the TyG-index and the time-varying effect of CMDs.

Therefore, based on the Kailuan study, this finding was aimed at examining the cumulative effects of the TyG-index, including its long-term level and change, on the risk of developing CMDs using up to 15 years of biennial longitudinal data.

## Subjects and methods

### Reasearch subjects

The Kailuan Study, which launched in July 2006 and remained ongoing, was an epidemiological survey that investigated and intervened in the risk factors for cardiovascular and cerebrovascular diseases among current and retired individuals at the Kailuan Group. The Kailuan Group is a massive-scale energy and chemical enterprise primarily dominated by coal mining products, where male employees make up a majority of the proportion. All the participants were followed up biennially by responding to validated questionnaires on demographics, health-related behaviors, and medical conditions, undergoing clinical examinations and laboratory tests. Detailed descriptions of the study design have been published (17, 18).

**Figure 1:**
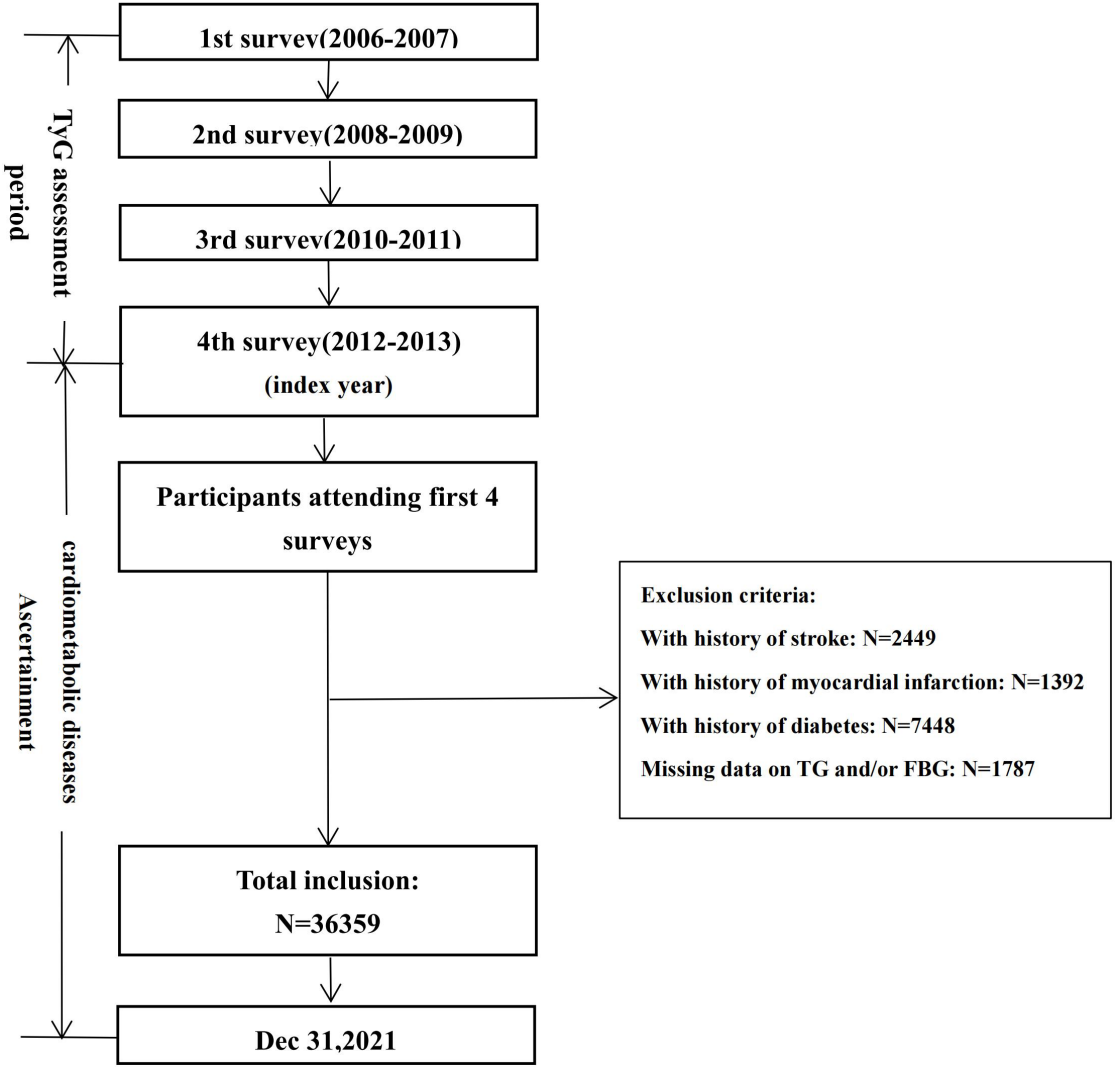
Flow chart of the current study.

Given the long-term level and change in the TyG-index, all 49435 subjects ≥18 years old or above underwent four health-checks consecutively from 2006–2007, 2008–2009, 2010–2011, and 2012-2013 (index year). For these remaining subjects, we excluded those who experienced a history of stroke (n=2449), myocardial infarction (n=1392), or diabetes (n=7748) and with missing data on triglyceride (TG) or fasting blood glucose (FBG) (n=1787) in or prior to 2012–2013. Ultimately, 36359 subjects were included to analyze the association between long-term level and change in the TyG-index with incident CMDs **(** **Figures 1**, **S1** **)**. The study was approved by the ethics committee of Kailuan General Hospital, and all the subjects gave written informed consent according to the guidelines of the Declaration of Helsinki.

## Data collection and definitions

### TyG-index calculation

The TyG-index was calculated as Ln [TG (mg/dl) ×FBG (mg/dl)/2] (19). The FBG and TG data were assayed using standard laboratory procedures in the central laboratory of the Kailuan General Hospital. Approximately 5 ml samples were extracted from the cephalic vein into an anticoagulant (EDTA) tube from 7:00–9:00 a.m. on the day of the health examination after overnight (>8 h) fasting. The FBG and serum TG were assessed using the hexokinase method (the coefficient of variation was <2%, and the upper limit of linearity was 33.3 mmol/L) and enzymatic colorimetric method, respectively. The hematological parameters of the blood were analyzed by an autoanalyzer (Hitachi, Tokyo, Japan). The corresponding kits were purchased from Zhongsheng North Control Biotechnology Co., Ltd. The professional quality controller regularly monitored these parameters.

### Average TyG-index and change of TyG-index were performed using the following formula:


$$①\;{\text{Average TyG index}}_{06-12}\text{= }\left({\text{TyG}}_{06}{\times \text{Time}}_{1-2}\right)\;+\;\left({\text{TyG}}_{08}{\times \text{Time}}_{2-3}\right){\text{ + (TyG}}_{10}{\times \text{Time}}_{3-4}{\text{)]/Time}}_{1-}4_{\;}$$

$$②\;\text{Average TyG index= [}\left({\text{TyG}}_{06}{\times \text{Time}}_{1-2}\right)\;+\;\left({\text{TyG}}_{08}{\times \text{Time}}_{2-3}\right){+\text{(TyG}}_{10}{\times \text{Time}}_{3-4}{\text{)+(TyG}}_{12}{\times \text{Time}}_{4-}\text{)]/Total Time}$$

$$③\;\text{Total TyG index change}=\;\left[\left({\text{TyG}}_{08}{-\text{TyG}}_{06}\right){\times \text{Time}}_{1-2}\right)\;+\;\left({\text{TyG}}_{10}{-\text{TyG}}_{08}\right){\times \text{Time}}_{2-3})\;+\;\left({\text{TyG}}_{12}{-\text{TyG}}_{10}\right){\times \text{Time}}_{3-4}{\text{)] / Time}}_{1-4}$$

$${\text{Time}}_{1-2}{=1}^{\text{st}}\text{measurement time }\left(2006\;-2007\right)\text{ to }2^{\text{nd}}\text{measurement time}\;(2008-2009);$$

$${\text{Time}}_{2-3\;=}2^{\text{nd}}\text{measurement time}\;(2008-2009)\;{to 3}^{\text{rd}}\text{measurement time}\;(2010-2011);$$

$${\text{Time}}_{3-4\;=}3^{\text{rd}}\text{measurement time }(2010-2011)\;{to 4}^{\text{th}}\text{measurement time}\;(2011-2012);$$

$${\text{Time}}_{1-4=}1^{\text{st}}\text{measurement time}\;(2006-2007)\;{to 4}^{\text{th}}\text{measurement time}\;(2011-2012);$$

$${\text{Time}}_{4-}{=4}^{\text{th}}\text{measurement time}\;(2011-2012)\;\text{ to end of follow}-\text{up;}$$

$${\text{Total Time }=\;1}^{\text{st}}\text{measurement time }(2006\;-\;2007)\text{ to end of follow}-\text{up.}$$

According to the grouping method described in the reference (20), the subjects were stratified by the average TyG-index into quartiles: Q1 group, <8.22 (as reference group), Q2 group, 8.22–8.53, Q3 group, 8.53–8.90, and Q4 group, ≥8.90. We further analyzed the impacts of the TyG-index change on CMDs, including the total TyG change (TyG_06_-TyG_12_), early TyG change (TyG_06_-TyG_08_), middle TyG change (TyG_08_-TyG_10_), and late TyG change (TyG_10_-TyG_12_), separately. We classified the total, early, middle, and late TyG changes into quintiles according to the quintiles of the TyG-index change: Q1 group<-0.14, -0.14≤Q2 group<-0.025, -0.025≤Q3 group<0.07 (as reference group), 0.07≤ Q4 group<0.19, and Q5 group≥0.19.

Additionally, we conducted latent variable modeling, which was implemented by a group-based approach with SAS Proc Traj (21), to identify four distinctive TyG trajectories: low-stable (7990, 22.0%), moderate-low stable (18802, 51.7%), moderate-high stable (8018, 22.1%), and high stable (1549, 4.3%). The TyG-index variability was calculated using the standard deviation (SD) and coefficient of variation (CV) across the four TyG measurements (in 2006–2007, 2008–2009, 2010–2011, and 2011-2012).

### Assessment of variability using the following formula:


$$\text{SD= }\sqrt{\frac{1}{n-1}\sum_{i-1}^{1}{\left(xi-x\right)}^{2}}$$

CV=(SD/MEAN)×100 %

### Assessment of outcomes

The first occurrence of CMDs event was the primary endpoint outcome during follow-up, including MI, stroke, or T2DM, as described previously (1). ICD codes from the Tenth Revisions (ICD‐10) were used to identify diagnoses of CMD (either MI: I21, stroke: I60-I63, or T2DM: E11). The database of CMDs diagnoses was ascertained by searching the Hospital Discharge Register of the 11 hospitals and the Municipal Social Insurance Institution, which was updated annually throughout the follow-up period. All suspected new‐onset CMDs events were reviewed by a panel of 3 physicians who scrutinized all pertinent medical records and further identified the diagnosis. Diagnostic evidence of MI included a history of clinical symptoms, changes in electrocardiography and elevated concentrations of cardiac enzymes (22), according to the WHO criteria, while that of stroke included focal neurofunctional deficit signs and symptoms, and neuroimaging examination (23). T2DM was diagnosed either by receiving hypoglycemic drugs, a self-reported history of diagnosed diabetes, or FBG ≥7.0 mmol/L, based on the 2020 American Diabetes Association Standards (24).

### Covariates

The demographics collected during this study included age, sex (male, female), lifestyle factors including alcohol consumption (never/ever, current), smoking (never/ever, current), education level (middle school and below, high school and above), physical activity, and salt intake level; medical history (hypertension, CMD); medication history (antihypertensive, hypoglycemic, or lipid-lowering drugs), and other information *via* face-to-face interview questionnaires. Standard exercise was defined as performing aerobic exercise ≥3 times/week for >30 minutes each time continuously. The standard salt diet was defined as 10 g/day of salt intake. Hypertension was defined based on either receiving medications for hypertension, a self-reported history of hypertension, or blood pressure ≥140/90 mmHg (25).

All laboratory tests, including high-density lipoprotein cholesterol (HDL-C), high sensitivity C-reactive protein (hs-CRP), low-density lipoprotein cholesterol (LDL-C), and total cholesterol (TC), were performed using an automatic biochemical analyzer (7600-020).

### Statistical analysis

Statistical analysis was performed with R 4.1.1 and SAS 9.4 (SAS Institute Inc., Cary, NC). The baseline data are displayed as the means ± standard deviation (X ± SD), numbers (percentage), or medians (P25, P75), where appropriate. The incidence rate of new-onset CMDs was calculated by dividing the number of events by the total follow-up period (per 1000 person-years).

Cox proportional regression models were applied to assess the relationships for CMDs within a given category as follows: (i) average TyG-index (quartile 1<8.22 as the reference group), (ii) TyG-index change (-0.025≤quintile 3<0.07 as the reference group), (iii) TyG-index trajectory (low-stable trajectory as the reference group), and (iv) TyG-index variability (TyG-CV: quartile 1<3.76, TyG-SD: quartile 1<0.22 as the reference group). All the Cox proportional hazards models were adjusted for sex, age, educational level, smoking, salt intake, physical activity, alcohol consumption, LDL-C, hs-CRP, eGFR, HDL-C, BMI, lipid-lowering drugs, and antihypertensive drugs as potentially relevant confounders. The missing covariates were imputed using a fully conditional specification method.

To account for the association between CMDs and the level of TyG-index change over time, we ran time-dependent Cox regression models to repeat the above (i) and (ii) categories of analysis in which the time-varying covariates were incorporated into the models. In addition, we also assessed the impacts on CMDs risk at the four time points (TyG_06_, TyG_08_, TyG_10_, and TyG_12_).

We performed analyses stratified by gender (male, female), BMI (BMI ≤ 28, BMI>28), and age (≤60, >60 years) to assess whether the effect was modified. Furthermore, a subgroup analysis was conducted on the participants after stratification by the direction of change in TyG index (increase, decrease). To minimize the influence of potential bias from reverse causality, we also performed sensitivity analyses that excluded the first 1 year of follow-up. Considering the potential survival bias, we assessed the cross-sectional correlation of the TyG-index at baseline, the average TyG-index, and the TyG-index change with the risk of CMDs at baseline by running logistic regression models. *P*<0.05 was considered statistically significant for the bilateral tests.

## Results

### Baseline characteristics

**Table 1** presents descriptive characteristics of the baseline demographic and biochemical profiles. Overall, 36359 subjects (mean age 53.11 ± 11.79 years old, male 73.87%) comprised the study population. We grouped the subjects according to the quartiles of the baseline TyG-index. When compared to the Q1 group, subjects in the other groups were more inclined to be more current smokers, current drinkers, and male, had a higher SBP, DBP, BMI, FBG, hs-CRP, TG, LDL-C, TC level, and a higher prevalence of hypertension (*P*<0.01).

**Table 1:** Baseline characteristics according to quartiles of baseline TyG index.

	Total	Quartile 1	Quartile 2	Quartile 3	Quartile 4	*P*
Participants	36359	9093	9084	9093	9089	
Age, year	53.11 ± 11.79	52.47 ± 12.34	53.58 ± 11.88	54.19 ± 11.56	52.22 ± 11.23	<0.01
Male, N (%)	26857 (73.87)	6228 (68.49)	6699 (73.75)	6787 (74.64)	7143 (78.59)	<0.01
SBP, mmHg	128.55 ± 18.28	124 ± 18.22	127.76 ± 18.14	130.60 ± 18.32	131.82 ± 17.46	<0.01
DBP, mmHg	82.97 ± 10.25	80.18 ± 10.03	82.42 ± 9.99	83.91 ± 10.20	85.37 ± 10.07	0.32
BMI, Kg/m²	24.99 ± 3.39	23.84 ± 3.17	24.62 ± 3.29	25.39 ± 3.33	26.11 ± 3.32	<0.01
FBG, mmol/L	5.25 ± 0.63	4.99 ± 0.58	5.17 ± 0.58	5.36 ± 0.60	5.50 ± 0.65	<0.01
TC, mmol/L	5.04 ± 0.98	4.62 ± 0.84	4.92 ± 0.88	5.20 ± 0.94	5.43 ± 1.03	<0.01
LDL-C, mmol/L	2.49 ± 0.83	2.24 ± 0.71	2.51 ± 0.76	2.66 ± 0.84	2.55 ± 0.94	<0.01
HDL-C, mmol/L	1.39 ± 0.39	1.54 ± 0.39	1.44 ± 0.35	1.36 ± 0.37	1.23 ± 0.39	<0.01
TG, mmol/L	1.21 (0.87-1.83)	0.68 (0.57-0.79)	1.04 (0.94-1.15)	1.45 (1.30-1.63)	2.56 (2.10-3.48)	<0.01
hs-CRP, mg/L	1.20 (0.59-2.30)	0.97 (0.49-1.90)	1.12 (0.50-2.35)	1.24 (0.61-2.38)	1.42 (0.75-2.60)	0.12
eGFR, [mL/(min·1.73²)]	92.53 (75.59-105.32)	95.17 (78.51-107.69)	90.52 (74.24-103.86)	91.62 (74.81-104.20)	92.86 (75.27-105.80)	<0.01
Current smoking, N (%)	11806 (32.47)	2553 (28.08)	2720 (29.94)	3011 (33.11)	3522 (38.75)	<0.01
Current drinking, N (%)	11010 (30.28)	2403 (26.43)	2482 (27.32)	2716 (29.87)	3409 (37.51)	<0.01
Physical exercisers, N (%)	25885 (71.19)	6246 (68.69)	6694 (73.69)	6546 (71.99)	6399 (70.40)	<0.01
Education level, N (%)						<0.01
High school diploma or below	26871 (73.90)	6472 (71.18)	6878 (75.72)	6862 (75.46)	6659 (73.26)	<0.01
High school diploma or above	9488 (26.10)	2621 (28.82)	2206 (24.28)	2231 (24.54)	2430 (26.74)	<0.01
Salt level, g/d						<0.01
<10	4389 (12.07)	1147 (12.61)	1016 (11.18)	1134 (12.47)	1092 (12.01)	<0.01
>10	31970 (87.93)	7946 (87.39)	8068 (88.82)	7959 (87.53)	7997 (87.99)	<0.01
Hypertension, N (%)	14766 (40.61)	2693 (29.62)	34373 (38.23)	4088 (44.96)	4512 (49.64)	<0.01
Antihypertensive treatment, N (%)	4906 (13.49)	775 (8.52)	1012 (11.14)	1425 (15.67)	1694 (18.64)	<0.01
Lipid-lowering treatment, N (%)	395 (1.09)	44 (0.48)	56 (0.62)	102 (1.12)	193 (2.12)	<0.01

P, comparison of baseline characteristics between different TyG index groups

SBP systolic blood pressure, DBP diastolic blood pressure, BMI body mass index, FBG fasting blood glucose, TC total cholesterol, LDL-C low-density lipoprotein cholesterol, HDL-C high-density lipoprotein cholesterol, hs-CRP high-sensitivity C reactive protein, TG triglyceride, eGFR estimated glomerular filtration rate.

TyG index: triglyceride glucose index.

### Average TyG-index and CMDs risk

During the mean observation period of 8.03 years, 4685 subjects were diagnosed with new-onset CMDs (including 1708 CVD events, 2772 diabetes events and 205 CVD and diabetes events). In addition, the cumulative incidence and the incidence rate for CMDs were 12.89% and 16.05 per 1000 person-years, respectively.

**Table 2** showes the association between incident CMDs and the average TyG-index. The multivariable-adjusted HRs for subjects in the Q2-Q4 groups were 1.64(1.47-1.83), 2.36(2.13-2.62), and 3.15(2.84-3.49), compared with those in the Q1 group. The associations of the average TyG-index with the CMDs risk became more significant compared with the primary analysis, after introducing confounders as time-varying covariates based on the time-dependent Cox regression models.

**Table 2:** Hazard ratios (95% CI) for risk of outcomes according to quartiles of Average TyG-index (1^st^ measurement to end of follow-up).

	Quartile 1	Quartile 2	Quartile 3	Quartile 4	*P* for trend
Multivariable adjusted HR (95% CI)					
Cases, N (%)	531(5.84)	942(10.36)	1399(15.39)	1813(19.94)	
Incidence, per1000 person-y	7.03	12.76	19.42	25.67	
Model.1	Reference	1.77(1.59-1.97)	2.65(2.40-2.93)	3.65(3.32-4.03)	0.01
Model.2	Reference	1.70(1.53-1.90)	2.50(2.26-2.77)	3.39(3.07-3.74)	0.01
Model.3a	Reference	1.64(1.47-1.83)	2.36(2.13-2.62)	3.15(2.84-3.49)	0.01
Time-dependent variables adjusted HR (95% CI)					
Cases, N (%)	531(5.84)	942(10.36)	1399(15.39)	1813(19.94)	
Incidence, per1000 person-y	7.03	12.76	19.42	25.67	
Model.1	Reference	1.80(1.69-1.92)	2.77(2.60-2.95)	3.71(3.50-3.94)	0.01
Model.2	Reference	1.78(1.67-1.91)	2.75(2.59-2.93)	3.72(3.50-3.95)	0.01
Model.3b	Reference	1.66(1.56-1.78)	2.44(2.29-2.60)	3.16(2.95-3.35)	0.01

Model 1:adjusted for age and sex;

Model 2:adjusted for age, sex, Smoking, Drinking, Education level, Salt status and Physical activity, BMI;

Model 3:adjusted for all the variables in model 2 and LDL-C, HDL-C, hs-CRP, eGFR, Antihypertensive treatment, Lipid-lowering treatment.

**Figure 2:**
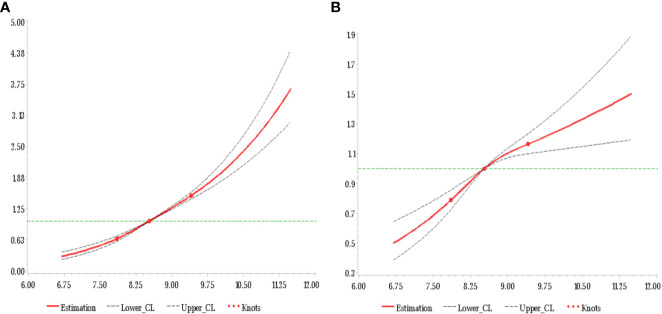
HR of CMDs and TyG-index at baseline adjusted for all covariates **(A)** and additional adjustment for average TyG-index up to baseline **(B)**.

One goal of the analysis was to explore the relative importance of the average TyG-index and that of the most recent TyG-index values. Even after adjustment for the baseline TyG-index, the average TyG-index was correlated with CMDs risk in the Q2 (HR 1.34, 95% CI 1.20-1.49), Q3 (HR 1.83, 95% CI 1.65-2.03), and Q4 (HR 2.39, 95% CI 2.14-2.67) groups compared with the Q1 group (**Table 3**). In contrast, there was no strong association between the risk of CMDs and the current baseline TyG-index following adjustment for the average TyG-index **(** **Figure 2** **)**.

**Table 3:** Hazard ratios (95% CI) for risk of outcomes according to quartiles of Average TyG-index (1^st^ measurement to 4^th^ measurement).

	Quartile 1	Quartile 2	Quartile 3	Quartile 4	P for trend
Cases, N (%)	576(6.34)	892(9.81)	1324(14.57)	1893(20.83)	
Incidence, per1000 person-y	7.62	12.06	18.31	26.99	
Model.1	Reference	1.53(1.38-1.70)	2.32(2.10-2.56)	3.49(3.17-3.83)	0.01
Model.2	Reference	1.48(1.34-1.65)	2.20(1.99-2.42)	3.24(2.95-3.57)	0.01
Model.3c	Reference	1.43(1.29-1.59)	2.06(1.86-2.28)	3.00(2.72-3.31)	0.01
Model.3d	Reference	1.36(1.22-1.51)	1.87(1.67-2.09)	2.51(2.20-2.87)	0.01
Model.3e	Reference	1.34(1.20-1.49)	1.83(1.65-2.03)	2.39(2.14-2.67)	0.01

Model 1: adjusted for age and sex;

Model 2: adjusted for age, sex, Smoking, Drinking, Education level, Salt status and Physical activity, BMI;

Model 3c:adjusted for all the variables in model 2 and LDL-C, HDL-C, hs-CRP, eGFR, Antihypertensive treatment, Lipid-lowering treatment.

Model 3d:adjusted for all the variables in model 2 and LDL-C, HDL-C, hs-CRP, eGFR, Antihypertensive treatment, Lipid-lowering treatment, tyg_06_.

Model 3e:adjusted for all the variables in model 2 and LDL-C, HDL-C, hs-CRP, eGFR, Antihypertensive treatment, Lipid-lowering treatment, tyg_12_.

When we assessed the HRs and 95% CIs for TyG_06_, TyG_08_, TyG_10_, and TyG_12_ separately, these results indicated that a high TyG level was positively related to an increased risk of subsequent incident CMDs at each time point (**Supplementary Table S1**).

### TyG-index change and CMDs risk

A Cox proportional hazards regression analysis, which was applied to explore the relationship between CMDs and the TyG-index change, is shown in **Table 4**. When compared to the stable group (0.025≤Quintile 3<0.07), we found a correlation between both loss and gain in TyG level and an increased CMDs risk. In the case of the total TyG-index change, the HRs (95% CI) for subjects in the Q1, Q2, Q4 and Q5 groups were 1.14 (1.02-1.27), 1.09 (0.99-1.20), 1.08 (0.98-1.19), and 1.14 (1.02-1.27), respectively, compared with the stable TyG-index group. Furthermore, for early, middle, and late changes in the TyG-index, the multivariable-adjusted HRs (95% CI) within the Q5 group compared with the reference group were 1.13 (1.00-1.27), 1.10 (0.98-1.23), and 1.12 (1.00-1.26), respectively. Essentially similar results were revealed according to the time-dependent Cox regression models.

**Table 4:** Hazard ratios (95% CI) for risk of outcomes according to quintiles of TyG index change.

	Cases, N (%)	Incidence, per 1000 person-y	Whole period (06-12)	Early period (06-08)	Middle period (08-10)	Late period (10-12)
Multivariable adjusted HR (95% CI)						
Quintile 1	867 (11.92)	14.81	1.14 (1.02-1.27)	1.15 (1.02-1.29)	1.09 (0.97-1.22)	1.09 (0.97-1.22)
Quintile 2	995 (13.68)	17.22	1.09 (0.99-1.20)	1.06 (0.92-1.23)	1.00 (0.87-1.15)	1.06 (0.92-1.22)
Quintile 3	953 (13.11)	16.42	Reference	Reference	Reference	Reference
Quintile 4	919 (12.64)	15.65	1.08 (0.98-1.19)	1.04 (0.90-1.21)	1.01 (0.87-1.16)	1.10 (0.96-1.27)
Quintile 5	951 (13.08)	16.16	1.14 (1.02-1.27)	1.13 (1.00-1.27)	1.10 (0.98-1.23)	1.12 (1.00-1.26)
Time-dependent variables adjusted HR (95% CI)						
Quintile 1	867 (11.92)	14.81	1.06 (0.99-1.14)	1.23 (1.14-1.32)	1.06 (0.99-1.13)	1.04 (0.97-1.11)
Quintile 2	995 (13.68)	17.22	1.11 (1.05-1.18)	1.16 (1.06-1.27)	1.03 (0.95-1.12)	1.01 (0.93-1.10)
Quintile 3	953 (13.11)	16.42	Reference	Reference	Reference	Reference
Quintile 4	919 (12.64)	15.65	1.11 (1.04-1.17)	1.11 (1.02-1.21)	0.99 (0.91-1.08)	1.05 (0.97-1.14)
Quintile 5	951 (13.08)	16.16	1.18 (1.10-1.26)	1.17 (1.09-1.26)	1.06 (0.99-1.13)	1.10 (1.03-1.18)

**Figure 3:**
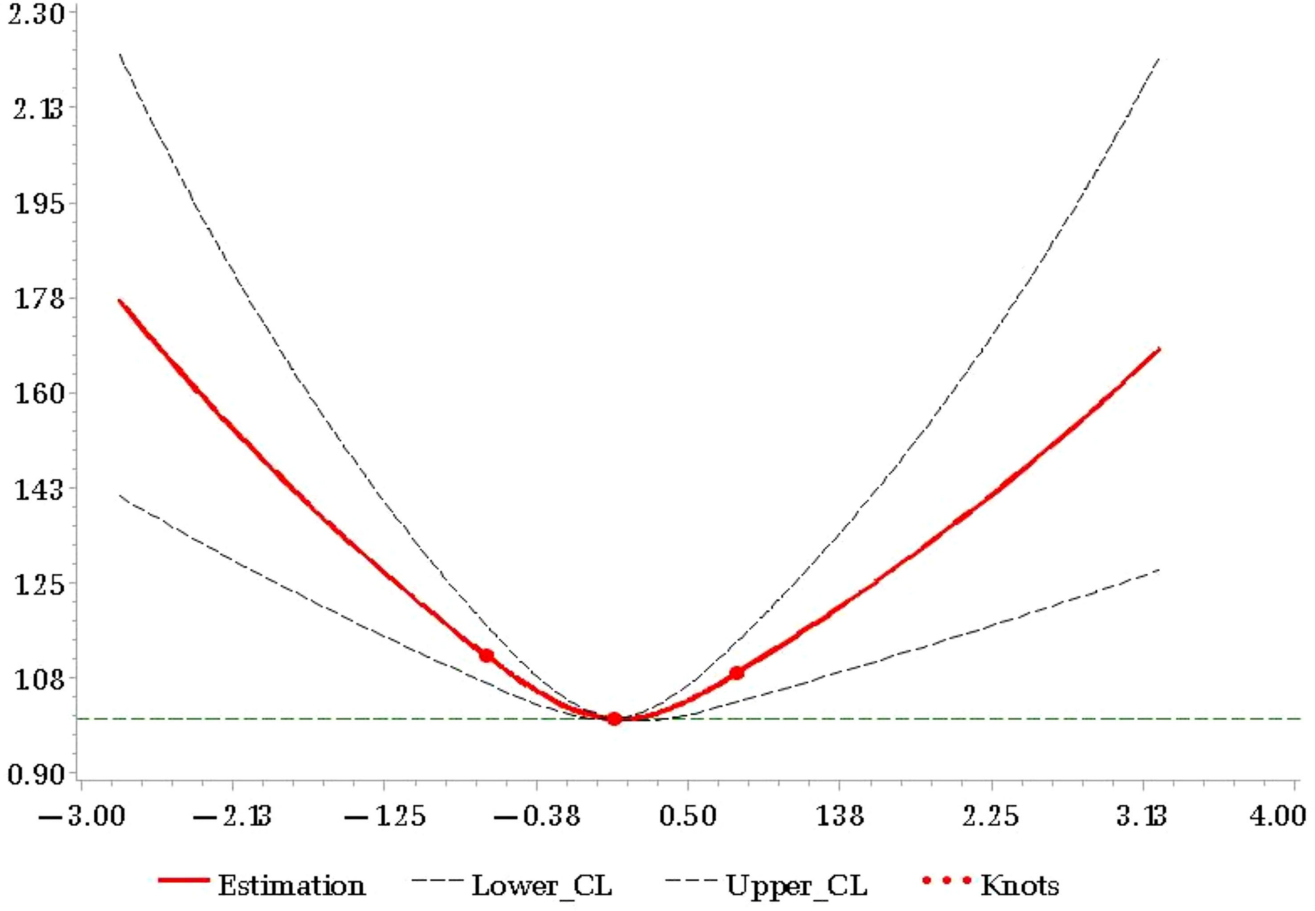
HR of CMDs and TyG-index change adjusted for all covariates.

Model:adjusted for age, sex, Smoking, Drinking, Education level, Salt status, Physical activity, BMI, LDL-C, HDL-C, hs-CRP, eGFR, Antihypertensive treatment, Lipid-lowering treatment,

**Figure 3** displays the association between the TyG-index change and CMDs, as fitted by restricted cubic splines (*P*_linearity_<0.001, *P*_non-linearity_=0.001), after adjustment for potential confounders. Furthermore, when considered as a continuous variable, the relationship between TyG-index change and CMDs risk followed a U-shaped curve.

### TyG-index trajectory, TyG-index variability and CMDs risk

As shown in **Supplementary Table S2**, for either the trajectory or variability in the TyG-index, the association between these variables and the risk of CMDs was assessed using the Cox proportional hazard model. When compared with the low-stable trajectory group, the risk of CMDs incidence in the high-stable trajectory group was highest (HR 3.86, 95% CI 3.35-4.45), followed by the moderate-high stable trajectory group (HR 2.95, 95% CI 2.65-3.29) and the moderate-low stable trajectory group (HR 1.82, 95% CI 1.65-2.01). For TyG-index variability, compared to those of the reference group, subjects with the highest degree of TyG-index variability (either TyG-SD or TyG-CV) were related to a higher CMDs risk (HR 1.13, 95% CI 1.04-1.22, HR 1.22, 95% CI 1.12-1.32, correspondingly).

### Hierarchical analyses and sensitivity analyses

After correction for the potential variables, the detailed results of the hierarchical analyses are presented in **Supplemental Tables S3-S6**. We found significant interactions between age (≤60, >60), gender (male, female), and BMI (BMI ≤ 28, BMI>28) in relation to the average TyG-index (*P*_Interaction_<0.05). Moreover, the relative risks for CMDs were likely to be higher among ≤60 years old, female, and non-obese individuals by categories of the average TyG-index. For the change in the TyG-index, no significant interactions were detected between potential risk factors for the risk of CMDs, such as age (≤60, >60), sex, and BMI (BMI ≤ 28, BMI>28). Using the intermediate group (Q3) as a reference, the HRs (95% CI) within ≤60 years old, males, and non-obese groups were 1.25 (1.09-1.44), 1.14 (1.01-1.29), and 1.17 (1.03-1.33), respectively. However, no statistically significant differences were observed in elderly individuals, female, and obese groups. **Supplementary Table S6** showed that whether increase or decrease in TyG index, the relationship between CMDs and change in TyG index is positively correlated with the magnitude of change.

In the sensitivity analyses, the results were consistent with the main analyses when a total of 330 CMDs cases were excluded after the first year of follow-up (**Supplementary Table S7**). The cross-sectional associations between the TyG-index and prevalent CMDs at baseline generally echoed the corresponding prospective associations (**Supplementary Table S8**). The results of the above sensitivity analyses demonstrated the good robustness of the association without reducing the estimate.

## Discussion

In the present research, the major discovery is that a long-term high TyG-index and a change in the TyG-index are independently important risk factors for CMDs and combine over time to increase the risk of having CMDs. Moreover, a high TyG-index in the early stage, even after accounting for the baseline TyG-index, exerted cumulative impacts on the development of CMDs. Moreover, an increased variability and longitudinal trajectory in the TyG-index were also correlated with an increased risk of CMD occurrence.

IR is widely known as a primary risk factor for T2DM and CVD, and associated with metabolic abnormalities (7, 8, 26). On account of the complex detection process and the clinical complexity, the hyperinsulinemic euglycemic glucose clamp (HEGC), which was considered as the gold standard for IR assessment (27). Additionally, homeostatic model assessment of insulin resistance (HOMA-IR), which is derived from fasting plasma glucose and fasting insulin, has been used as an alternative indictor for defining IR (28). Its measurement of insulin is consistently limited in clinical practice. Moreover, although Mounting evidence indicate that TyG index is correlate with the HOMA-IR, TyG index has high sensitivity for recognizing insulin resistance compared with the HOMA-IR (29). Thus, we focused on the TyG index due to its characteristics of simplicity and cost-efficient in this research.

Over the long-term follow-up (up to 8 years), we found that elevated TyG level are correlated with an increased risk of developing CMDs. Although the abovementioned idea has been demonstrated by previous studies (14, 15, 30–34), the measurement of the circulating TyG-index, which was measured only once, makes it impossible to reflect on the long-term influence between the TyG level and the development of CMDs. A meta-analysis combining 5 prospective studies showed a positive association between atherosclerotic cardiovascular disease (ASCVD) events and the TyG-index (33). A high TyG level has been reported to be closely correlated with T2DM in a prospective cohort study in Singapore (34). Our research further expands the pre-existing knowledge to explore the cumulative effect of the long-term TyG-index on the development of CMD occurrence in the Kailuan Study. The subjects in the fourth quartile had a 3.15-fold higher risk of developing CMDs than those in the first quartile. After correction for TyG_06_ and TyG_12_, when compared to the uncorrected data (HR: 3.00, 95% CI: 2.72-3.31), the HRs (95% CI) of the average TyG_06-12_ index on CMDs were 2.51 (2.20-2.87) and 2.39 (2.14-2.67), respectively. This result suggests that the effect of the long-term TyG level on CMDs was independent and superior to the single point measurement of the TyG level. Moreover, when using updated confounders within the follow-up period, a significant short-term increase in the risk of CMDs with elevated TyG level was observed in this finding.

Given that TG and FBG, both of which are utilized to calculate the TyG-index, have been considered time-varying exposures. Of note, due to a single measurement of the TyG-index, this characteristic cannot be avoided for the potential regression dilution bias (35). It has been proposed that the above limitations and methodological insufficiencies can be addressed by assessing the influence of TyG-index change on the outcomes. Two cohort studies performed by Zhang et al (36)and Wang et al (16), demonstrated an association between change in the TyG-index and the risk of T2DM and CVD, respectively. However, those studies only considered the magnitude of the TyG-index change and not the direction. Compared with the stable TyG-index, our findings revealed that the effects of TyG level gain and loss were both directly correlated with a 14% increase in the risk of developing CMDs. This result suggests that the TyG-index change is positively related to the risk of CMDs occurrence, whether they are positive or negative. The results of this study were in accordance with previous studies on the effect of TyG-index gain on CMDs development; rather, the effect of TyG level loss has not been reported in the relevant literature, currently.

As a surrogate for identifying IR, the results from this research seem counterintuitive given that TyG-index loss was associated with CMDs. First, we found that the average TyG level was the highest in decrease group (Quintile 1) compared to other groups from the baseline table of change in TyG index (**Table S9**). Thus, it is reasonable to assume that, even if the current “ideal state” exists, long exposure to a high TyG-index during the early period of the observation window may increase the possibility of residual risk of CMDs before the TyG-index level decreases. Second, considering the fact that the determinants of TyG are the fasting glucose and triglycerides, similar conclusions have been reached in **Table S9**. For triglycerides, a clinical, randomized controlled trial (37) concluded that among patients with mild-to-moderate hypertriglyceridemia, the incidence of cardiovascular events was not lower among those who received pemafibrate than among those who received placebo, although pemafibrate lowered triglyceride level. Also, the”metabolic memory”effect may cause an increasing risk of CMDs when FBG is at a high level for a prolonged period of time in early life (38). Furthermore, owing to the possibility of reverse causality, participants in the TyG level loss category were more inclined to be at high risk for having a CMDs, such as drinkers and smokers **(** **Table S9** **)**, who have greater odds of receiving health education about CMDs prevention, adherence to medication and consciously lowering TyG levels. Notably, although we have tried to adequately adjust for confounders, there are still other unconsidered or uncontrollable factors, such as some insidious diseases, that may cause unintentional TyG level loss during the protracted or chronic course of the disease. For the above reasons, it can be said that the true association between TyG level loss and CMDs risk may be distorted among subjects with relatively high TyG levels in the early period of life, which might cause the spurious association that the lower TyG-index increases CMDs risk.

Provocatively, we found that the TyG-index change in the early stage was more strongly associated with the occurrence of CMDs after dividing the approximately 8-year observation window into three stages: early, intermediate and late. All these signs indicate that in a clinical setting, more attention should be given to individuals with significant TyG-index change. For high‐risk individuals with an elevated TyG-index, early prevention and control added strong evidence-based support for the primary prevention of CMDs.

It was recently noted that the variability and longitudinal trajectory in the TyG-index, both regarded as novel and important indicators for outcome events, had strong predictive effects for cardiovascular diseases (39–42). With either variability or longitudinal trajectory in the TyG-index, the effects on CMDs risk in this research are consistent with a previous study that found those indicators (variability and trajectory) to be associated with CMDs risk. In the occurrence and development of CMDs, our findings reveal that long-term level and change in the TyG-index play an important role, further emphasizing the need to maintain TyG level homeostasis in CMDs prevention. From another long-term perspective, closely monitoring the TyG-index, could elicit the maximum potential health benefit. Simultaneously, our findings also provide novel ideas for a clinical solution to screen high-risk CMDs individuals.

Several potential mechanisms link the sustained TyG-index (insulin resistance) and CMDs. Endothelial dysfunction is considered a key pathophysiological process in atherosclerosis initiation and progression (43, 44). Basic experiments indicated that the impairment of IR can mediate the injury and functional alterations of smooth muscle cells, mononuclear macrophages, and vascular endothelial cells by interfering with the insulin signaling pathway, which in turn leads to endothelial dysfunction (44). Moreover, the prolonged hyperglycemia state induced by IR may result in oxidative stress, coagulation dysfunction and the chronic inflammatory reaction of the human body, which are considered to be the driving factors in the development of dyslipidemia, metabolic syndrome and CMDs (45). Lastly, individuals with a long-term high TyG-index tend to experience complications with other traditional risk factors and comorbidities, such as smoking, drinking, and hypertension, which significantly increases the risk of CMDs. Although adjusting for the above confounders, it might not completely eliminate effects that are biologically meaningful. To date, because addressing the power of all of the relevant questions is limited, further basic research is needed to elucidate the pathophysiological significance of this finding.

Our findings have salient public health and clinical implications regarding the prevention of CMDs event development. First, the importance of early well-controlled and prevention of the TyG-index among young and middle-age groups. Second, either long-term changes in TyG level or increased variability in the TyG-index increased the risk of developing CMDs events. Thus, health education, physical exercise and a balanced diet could be regarded as some measures to maintain the long-term TyG-index at the “ideal level”. Third, along with the current TyG-index, we cannot ignore the importance of considering the history of TyG levels when assessing CMDs risks. Therefore, in terms of clinical application, the dynamic monitoring of the TyG-index and refined information on electronic medical records (including change and variability of the TyG-index) all shed new light on the prevention of CMDs.

This research has several strengths. The primary strength is that we conducted long-term follow-up of a large community-based population based on the Kailuan study to obtain more comprehensive biological information. Second, this is the first massive-scale prospective study to explore the relationships between long-term TyG-index levels and changes and incident CMDs. Moreover, several limitations of the current study must be acknowledged. First, the sex distribution of the study population was uneven (male 73.87%), while the characteristics and mechanisms of CMD occurrence may differ between males and females. Second, longitudinal data are naturally hampered by missing data at different time points according to an 8-year observation window. Third, due to the observational nature of the study, the association between long-term level and change in the TyG-index may not necessarily reflect any causal link. Fourth, although the diagnosis of diabetes in this study took into account the medical history and use of glucose-lowering drugs during the follow-up time, a proportion of the population in this study was still diagnosed based on a single measurement of FBG without the oral glucose tolerance test or detection of glycated hemoglobin levels, thus possibly underestimating the prevalence of T2DM as well as CMDs. However, this consideration is widely applied in many large-scale epidemiological studies at home and abroad, such as the Framingham study.

## Conclusion

To summarize, an increased CMDs risk was associated with long-term elevated level and change in the TyG-index, and even the related risk of CMDs from exposure during the early stage tended to be higher. This finding strongly underlined the importance of tracking the long-term maintenance of an appropriate TyG-index within the desirable range over time to prevent the development of CMDs effectively.

## Data availability statement

The raw data supporting the conclusions of this article will be made available by the authors, without undue reservation.

## Ethics statement

The study was performed according to the guidelines of the Helsinki Declaration and was approved by the Ethics Committee of Kailuan General Hospital (approval number:2006-05). All participants agreed to take part in the study and provided informed written consent.

## Author contributions

WX wrote the main manuscript text and conceived and designed the study. LGao, HZ, LGuo, JL, HL, JS, and AX contributed to acquisition of data, analysis and interpretation of data and revision of the drafting of the manuscript. YW and SW performed the manuscript review. All authors contributed to the article and approved the submitted version.
