# Supplementary material for: Association of long-term triglyceride-glucose index level and change with the risk of cardiometabolic diseases
Source: Front Endocrinol (Lausanne). 2023 Mar 30;14:1148203. doi: 10.3389/fendo.2023.1148203 (PMC10098344; doi:10.3389/fendo.2023.1148203)
Supplement: Supplementary file 1 [file DataSheet_1.pdf]

Supplementary Material

Association of long-term triglyceride-glucose index level and change with the risk of cardiometabolic diseases

Wenqi Xu[1, 2], Haiyan Zhao[1], Lishu Gao[3], Lu Guo[1, 2], jianrong Liu[1], haixia Li[1], Junyan Sun[1], Aijun Xing[1], Shuohua Chen[1], Shouling Wu[1], Yuntao Wu[1]*

***Correspondence:** wyt0086@163.com

Table S1. Hazard ratios for CMDs according to categories of TyG-index at each time point.

	TyG06			TyG08			TyG10			TyG12		
TyG	Events	HR	95% CI	Events	HR	95% CI	Events	HR	95% CI	Events	HR	95% CI
Q1	750	1	Reference	718	1	Reference	595	1	Reference	661	1	Reference
Q2	1019	1.41	(1.28-1.56)	860	1.34	(1.22-1.48)	971	1.45	(1.31-1.60)	1001	1.37	(1.24-1.51)
Q3	1269	1.70	(1.54-1.87)	1530	1.74	(1.58-1.91)	1443	1.89	(1.71-2.08)	1250	1.62	(1.47-1.79)
Q4	1647	2.47	(2.25-2.72)	1577	2.32	(2.11-2.55)	1676	2.49	(2.26-2.74)	1773	2.37	(2.15-2.60)

Model：adjusted for age, sex, Smoking, Drinking, Education level, Salt status, Physical activity, BMI, LDL-C, HDL-C, hs-CRP, eGFR, Antihypertensive treatment, Lipid-lowering treatment.

Table S2. Hazard ratios for CMDs by TyG-index trajectories and TyG-index variability

	Model.1	Model.2	Model.3
TyG index trajectories			
Low-stable	Reference	Reference	Reference
Moderate-low stable	2.02（1.83-2.23）	1.93（1.75-2.13）	1.82（1.65-2.01）
Moderate-high stable	3.48（3.14-3.86）	3.23（2.91-3.59）	2.95（2.65-3.29）
High stable	4.59（4.01-5.26）	4.20（3.67-4.82）	3.86（3.35-4.45）
TyG index variability			
TyG-CV			
Quartile 1	Reference	Reference	Reference
Quartile 2	1.05（0.97-1.14）	1.05（0.97-1.14）	1.06（0.98-1.15）
Quartile 3	1.03（0.95-1.12）	1.03（0.95-1.12）	1.04（0.96-1.13）
Quartile 4	1.20（1.11-1.30）	1.19（1.10-1.29）	1.22（1.12-1.32）
TyG-SD			
Quartile 1	Reference	Reference	Reference
Quartile 2	1.07（0.98-1.16）	1.06（0.98-1.15）	1.07（0.98-1.16）
Quartile 3	1.13（1.04-1.23）	1.12（1.03-1.22）	1.13（1.04-1.22）
Quartile 4	1.35（1.25-1.47）	1.33（1.23-1.44）	1.33（1.22-1.44）

Adjusted for age, sex, Smoking, Drinking, Education level, Salt status, Physical activity, BMI, LDL-C, HDL-C, hs-CRP, eGFR, Antihypertensive treatment, Lipid-lowering treatment.

Table S3. Hazard ratios for outcome by average TyG-index and total TyG-index change stratified by age at 60 years

	< 60 years	≥ 60 years	
Average TyG index			<0.001
Quartile 1	Reference	Reference	
Quartile 2	1.79（1.54-2.07）	1.48（1.26-1.74）	
Quartile 3	2.91（2.53-3.34）	1.75（1.50-2.05）	
Quartile 4	3.87（3.37-4.45）	2.21（1.88-2.61）	
TyG index change			0.33
Quintile 1	1.13（0.98-1.30）	1.10（0.91-1.32）	
Quintile 2	1.14（1.01-1.28）	1.02（0.87-1.19）	
Quintile 3	Reference	Reference	
Quintile 4	1.14（1.01-1.28）	0.99（0.85-1.15）	
Quintile 5	1.25（1.09-1.44）	0.93（0.78-1.12）	

Model：adjusted for age, sex, Smoking, Drinking, Education level, Salt status, Physical activity, BMI, LDL-C, HDL-C, hs-CRP, eGFR, Antihypertensive treatment, Lipid-lowering treatment

Table S4. Hazard ratios for outcome by average TyG-index and total TyG-index change stratified by gender

	Women	Men	
Average TyG index			<0.01
Quartile 1	Reference	Reference	
Quartile 2	2.01（1.54-2.61）	1.57（1.40-1.77）	
Quartile 3	3.11（2.40-4.02）	2.22（1.98-2.49）	
Quartile 4	4.70（3.62-6.12）	2.89（2.58-3.23）	
TyG index change			0.16
Quintile 1	1.05（0.80-1.39）	1.15（1.02-1.30）	
Quintile 2	1.15（0.93-1.43）	1.08（0.97-1.20）	
Quintile 3	Reference	Reference	
Quintile 4	0.92（0.74-1.14）	1.13（1.02-1.26）	
Quintile 5	1.12（0.87-1.43）	1.14（1.01-1.29）	

Model：adjusted for age, sex, Smoking, Drinking, Education level, Salt status, Physical activity, BMI, LDL-C, HDL-C, hs-CRP, eGFR, Antihypertensive treatment, Lipid-lowering treatment

Table S5. Hazard ratios for outcome by average TyG-index and total TyG-index change stratified by BMI

	BMI≤28	BMI>28	
Average TyG index			<0.05
Quartile 1	Reference	Reference	
Quartile 2	1.70（1.51-1.92）	1.27（0.98-1.65）	
Quartile 3	2.45（2.18-2.74）	1.82（1.43-2.31）	
Quartile 4	3.34（2.98-3.75）	2.29（1.80-2.91）	
TyG index change			0.54
Quintile 1	1.19（1.04-1.35）	1.07（0.85-1.35）	
Quintile 2	1.11（1.00-1.24）	1.07（0.89-1.30）	
Quintile 3	Reference	Reference	
Quintile 4	1.06（0.95-1.18）	1.12（0.93-1.34）	
Quintile 5	1.17（1.03-1.32）	1.06（0.85-1.32）	

Model：adjusted for age, sex, Smoking, Drinking, Education level, Salt status, Physical activity, BMI, LDL-C, HDL-C, hs-CRP, eGFR, Antihypertensive treatment, Lipid-lowering treatment

**Table S6.** Hazard ratios for outcome by TyG-index change stratified by the direction of change			
	Model 1	Model 2	Model 3
decrease in TyG index (N=16451)			
Quartile 1	Reference	Reference	Reference
Quartile 2	1.07（0.95-1.21）	1.07（0.95-1.21）	1.08（0.96-1.22）
Quartile 3	1.10（0.97-1.24）	1.10（0.97-1.24）	1.10（0.98-1.25）
Quartile 4	1.14（1.01-1.29）	1.14（1.01-1.28）	1.17（1.04-1.32）
Per 1 SD	1.06（1.01-1.10）		
increase in TyG index (N=19908)			
Quartile 1	Reference	Reference	Reference
Quartile 2	1.05（0.94-1.18）	1.06（0.95-1.19）	1.05（0.94-1.18）
Quartile 3	1.03（0.92-1.15）	1.03（0.92-1.16）	1.03（0.92-1.15）
Quartile 4	1.17（1.05-1.31）	1.16（1.04-1.30）	1.13（1.01-1.26）
Per 1 SD	1.04(1.00-1.08)		

Model：adjusted for age, sex, Smoking, Drinking, Education level, Salt status, Physical activity, BMI, LDL-C, HDL-C, hs-CRP, eGFR, Antihypertensive treatment, Lipid-lowering treatment

Table S7. Excluding participants with incident CMDs occurring within the first year of follow-up(N=36029)

	Model 1	Model 2	Model 3
Average TyG index			
Quartile 1	Reference	Reference	Reference
Quartile 2	1.78（1.60-1.99）	1.72（1.54-1.92）	1.66（1.48-1.85）
Quartile 3	2.69（1.60-1.99）	2.54（2.29-2.81）	2.40（2.16-2.67）
Quartile 4	3.73（3.38-4.13）	3.46（3.13-3.83）	3.22（2.90-3.58）
TyG index change			
Quintile 1	1.15（1.04-1.26）	1.15（1.04-1.26）	1.13（1.01-1.27）
Quintile 2	1.12（1.02-1.23）	1.12（1.02-1.23）	1.11（1.01-1.22）
Quintile 3	Reference	Reference	Reference
Quintile 4	1.09（0.99-1.20）	1.09（0.99-1.20）	1.09（0.99-1.20）
Quintile 5	1.18（1.07-1.29）	1.17（1.06-1.28）	1.16（1.04-1.30）

Model：adjusted for age, sex, Smoking, Drinking, Education level, Salt status, Physical activity, BMI, LDL-C, HDL-C, hs-CRP, eGFR, Antihypertensive treatment, Lipid-lowering treatment

Table S8. Odds ratios (OR) and 95% CI for prevalent CMDs at baseline (2010-2012), by categories of TyG index at baseline, average TyG and TyG change

	OR_a	95% CI	OR_b	95% CI	OR_C	95% CI
TyG at baseline						
Quartile 1	1	Reference	1	Reference	1	Reference
Quartile 2	1.53	（1.40-1.67）	1.51	（1.38-1.64）	1.49	（1.37-1.63
Quartile 3	2.62	（2.41-2.84）	2.57	（2.36-2.78）	2.51	（2.31-2.73
Quartile 4	6.78	（6.27-7.32）	6.61	（6.11-7.15）	6.40	（5.90-6.95
Average TyG						
Quartile 1	1	Reference	1	Reference	1	Reference
Quartile 2	1.79	（1.62-1.97）	1.76	（1.60-1.94）	1.76	（1.59-1.94）
Quartile 3	3.89	（3.56-4.26）	3.82	（3.50-4.18）	3.78	（3.45-4.15）
Quartile 4	11.20	（10.28-12.21）	11.01	（10.09-12.00）	10.79	（9.86-11.81）
TyG change						
Quintile 1	1.77	（1.65-1.91）	1.79	（1.66-1.93）	1.82	（1.69-1.960）
Quintile 2	1.05	（0.98-1.14）	1.06	（0.98-1.15）	1.06	（0.98-1.15）
Quintile 3	1	Reference	1	Reference	1	Reference
Quintile 4	1.08	（1.00-1.17）	1.08	（1.00-1.17）	1.06	（0.98-1.15）
Quintile 5	1.60	（1.49-1.73）	1.59	（1.48-1.72）	1.55	（1.44-1.67）

ORa: adjusted for age and sex at baseline.

ORb: adjusted for age, sex, Smoking, Drinking, Education level, Salt status and Physical activity, BMI;

ORc：adjusted for all the variables in model 2 and LDL-C, HDL-C, hs-CRP, eGFR, Antihypertensive treatment, Lipid-lowering treatment.

Table S9. Baseline characteristics according to Quintile of change TyG index

	Total	Quintile 1	Quintile 2	Quintile 3	Quintile 4	Quintile 5	*P*
Participants	36359	7271	7272	7272	7272	7272	
Age, year	53.11±11.79	53.07±11.45	53.63±11.70	53.75±11.98	53.31±11.88	51.82±11.82	<0.01
Male, N (%)	26857（73.87）	5893（81.05）	5414（74.45）	5239（72.04）	5109（70.26）	5202（71.53）	<0.01
BMI, Kg/m²	24.99±3.39	24.83±3.32	24.84±3.45	24.93±3.36	25.04±3.40	25.31±3.38	<0.01
Average TyG	8.57±0.52	8.77±0.51	8.57±0.49	8.50±0.50	8.48±0.52	8.49±0.54	<0.01
Physical exercisers, N (%)	25885（71.19）	5546（76.28）	5325（73.23）	5095（70.06）	4937（67.89）	4982（68.51）	<0.01
Current smoking, N (%)	11806（32.47）	2458（33.81）	2271（31.23）	2290（31.49）	2320（31.90）	2467（33.92）	<0.01
Current drinking, N (%)	11010（30.28）	2255（31.01）	2023（27.82）	2179（29.96）	2169（29.83）	2384（32.78）	<0.01
Hypertension, N (%)	14766（40.61）	2888（39.72）	2896（39.82）	2919（40.14）	2993（41.16）	3070（42.22）	<0.01
Antihypertensive treatment, N (%)	4906（13.49）	895（12.31）	957（13.16）	985（13.55）	1049（14.43）	1020（14.03）	<0.01
Lipid-lowering treatment, N (%)	395（1.09）	90（1.24）	71（0.98）	78（1.07）	78（1.07）	78（1.07）	<0.01
FBG_{06}	5.00±0.64	5.16±0.66	5.07±0.61	5.0±0.61	4.91±0.61	4.84±0.63	<0.01
FBG_{08}	5.16±0.60	5.22±0.60	5.18±0.60	5.16±0.59	5.15±0.59	5.11±0.59	<0.01

Continue S9

	Total	Quintile 1	Quintile 2	Quintile 3	Quintile 4	Quintile 5	*P*
FBG_{10}	5.21±0.58	5.25±0.59	5.21±0.58	5.20±0.57	5.20±0.58	5.20±0.59	<0.01
FBG_{12}	5.25±0.63	5.06±0.64	5.18±0.62	5.27±0.61	5.34±0.61	5.42±0.63	<0.01
TG_{06}	1.21(0.85-1.80)	1.84(1.23-3.16)	1.29(0.98-1.81)	1.15(0.84-1.60)	1.05(0.75-1.50)	0.94(0.67-1.36)	<0.01
TG_{08}	1.26(0.86-1.76)	1.35(0.92-1.83)	1.25(.86-1.66)	1.21(0.83-1.67)	1.20(0.82-1.73)	1.29(0.87-1.92)	<0.01
TG_{10}	1.24(0.89-1.82)	1.30(0.95-1.87)	1.20(0.88-1.71)	1.19(0.85-1.69)	1.22(0.87-1.79)	1.34(0.92-2.07)	<0.01
TG_{12}	1.21(0.87-1.83)	0.95(0.69-1.25)	1.07(0.78-1.44)	1.18(0.86-1.64)	1.37(0.97-1.99)	1.90(1.30-3.00)	<0.01
TyG_{06}	8.53±0.62	9.00±0.64	8.60±0.52	8.46±0.53	8.35±0.54	8.23±0.57	<0.01
TyG_{08}	8.56±0.61	8.63±0.63	8.54±0.58	8.51±0.58	8.53±0.61	8.60±0.65	<0.01
TyG_{10}	8.60±0.61	8.66±0.62	8.55±0.58	8.54±0.58	8.57±0.60	8.67±0.66	<0.01
TyG_{12}	8.59±0.62	8.25±0.52	8.60±0.52	8.53±0.53	8.70±0.54	9.08±0.65	<0.01
