# Supplementary figures and images for: Association of long-term triglyceride-glucose index level and change with the risk of cardiometabolic diseases
Source: Front Endocrinol (Lausanne). 2023 Mar 30;14:1148203. doi: 10.3389/fendo.2023.1148203 (PMC10098344; doi:10.3389/fendo.2023.1148203)

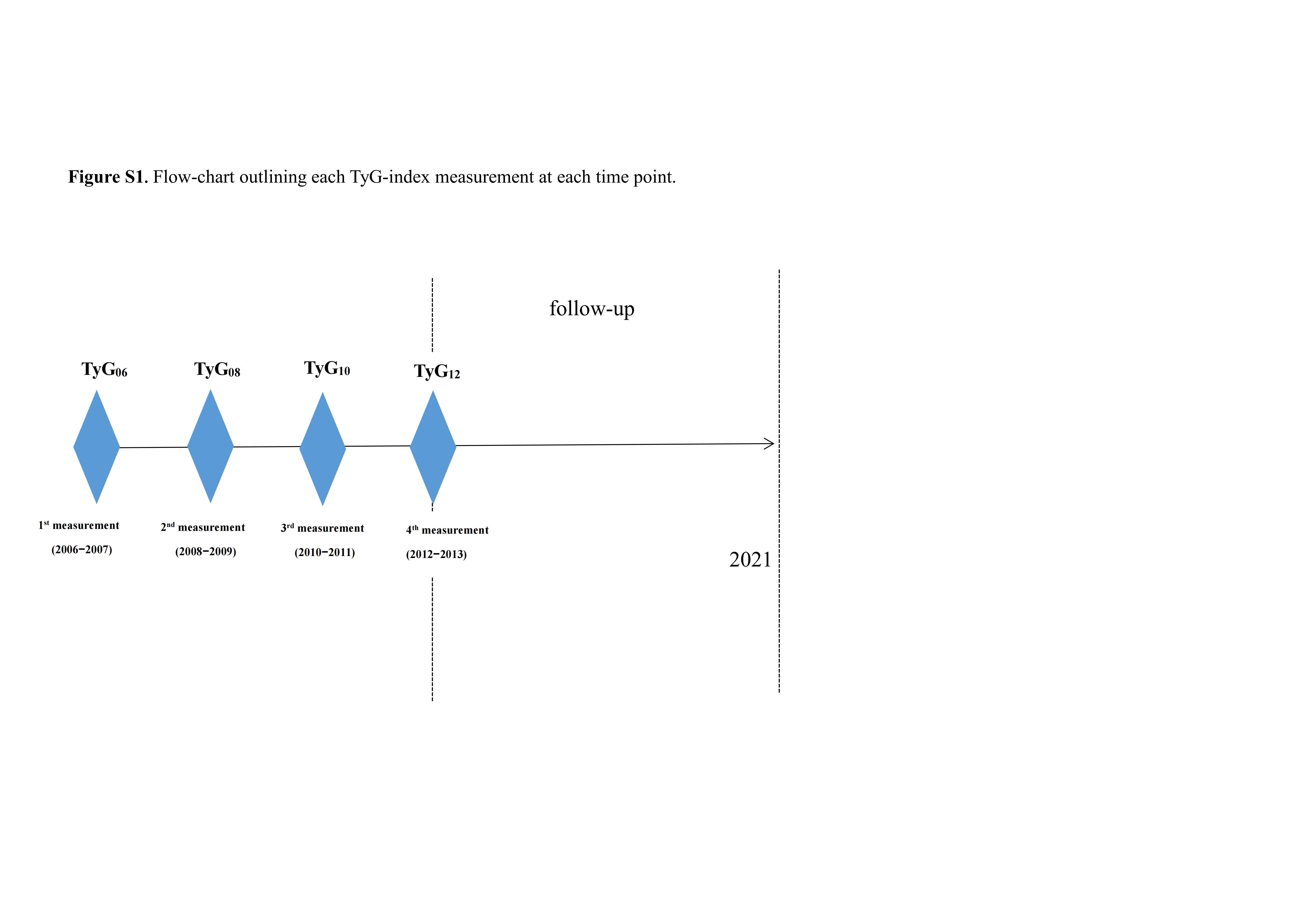

Supplement: Supplementary file 2 [file Image_1.jpeg]

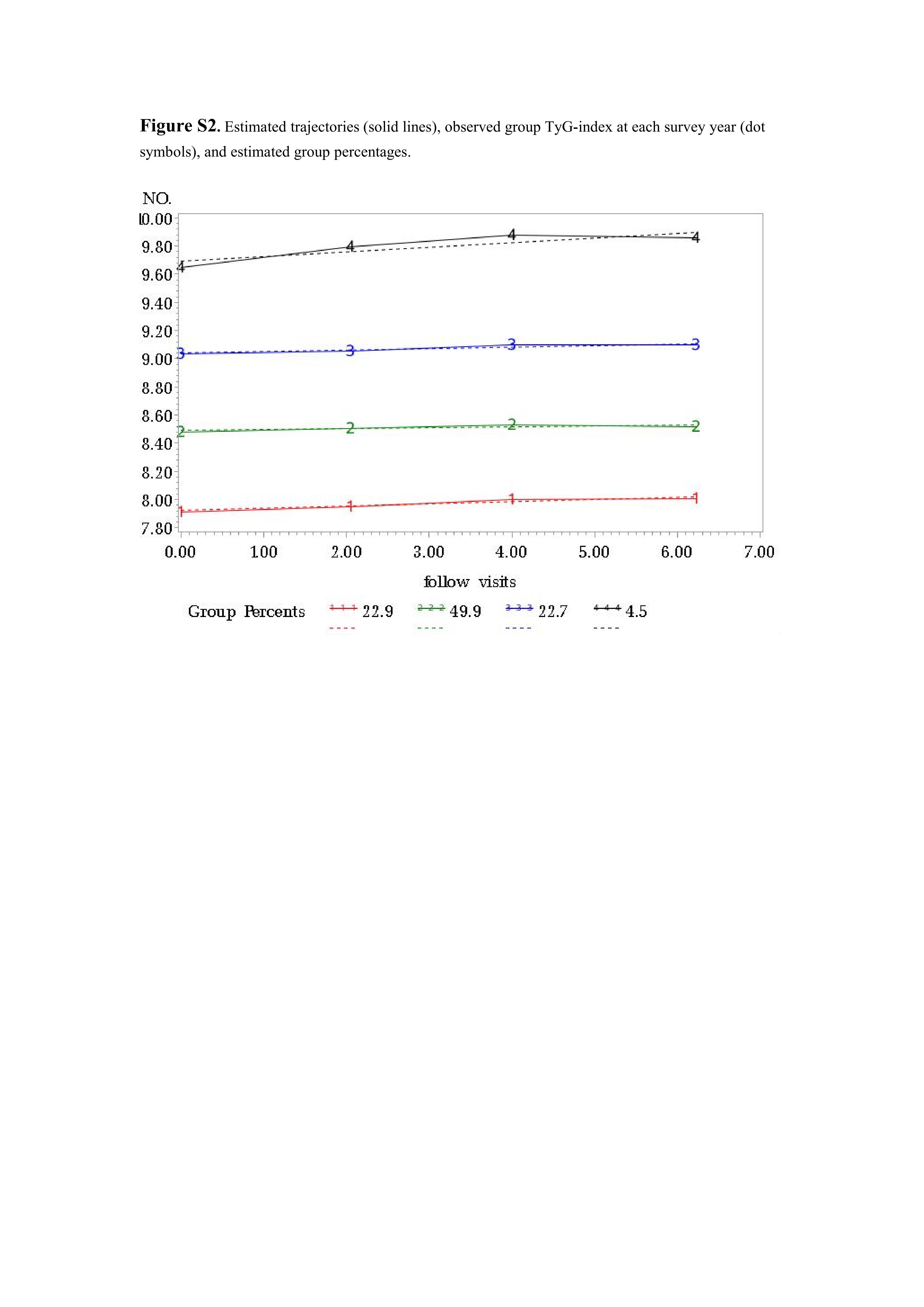

Supplement: Supplementary file 3 [file Image_2.jpeg]
